# Quality of Sleep in the Saudi Population During the Holy Month of Ramadan

**DOI:** 10.7759/cureus.50897

**Published:** 2023-12-21

**Authors:** Khalid M Al Harbi, Lubna Saleh Alsaleem, Abdulrahman I Alsaidan, Bader S Almalki, Rayan Qutub, Yousef Alammari

**Affiliations:** 1 College of Medicine, ‏Imam Mohammad Ibn Saud Islamic University, Riyadh, SAU; 2 College of Medicine, Imam Mohammad Ibn Saud Islamic University, Riyadh, SAU; 3 Department of Surgery, Imam Mohammad Ibn Saud Islamic University, Riyadh, SAU; 4 Department of Internal Medicine, Imam Mohammad Ibn Saud Islamic University, Riyadh, SAU

**Keywords:** sleep hygiene index, sleep quality scale, saudi population, ramadan, sleep hygiene, sleep quality

## Abstract

Background

The holy month of Ramadan carries a massive change in a Muslim’s lifestyle. During these 30 days of all-day fasting, people in Saudi Arabia prefer staying up all night and sleeping in the daytime, thus highly impacting their regular sleeping schedule, with a sizable effect on the quality of sleep. This study aimed to measure the quality of sleep and sleep hygiene practices in the Saudi population.

Methodology

This cross-sectional study was conducted among the Saudi population during the holy month of Ramadan. A self-administered questionnaire was sent to the targeted population using social media platforms. Our questionnaire included demographic data, the Sleep Quality Scale (SQS) to measure sleep quality, and the Sleep Hygiene Index (SHI) to measure sleep hygiene practices.

Results

Of the 386 participants, 71.2% were females, and 64.5% were single. The total mean SQS score was 35.8 (SD = 10.9) out of 84 points, while the total mean SHI score was 19.8 (SD = 8.61) out of 52 points. Overall, 63.5% of the respondents had poor sleep hygiene practices. Significant factors for increased SHI included being unmarried, a student, not the main provider in the family, not a parent, and earning less than 5,000 SAR per month while having more than six individuals in the family was a significant factor for increased SQS.

Conclusions

During the month of Ramadan, poor sleep quality and inadequate sleep hygiene practices were common among the Saudi population. A significant risk factor for poor sleep quality was having more than six individuals at home while being unmarried, a student, not being the family’s main provider, and earning less than 5,000 SAR per month were the significant risk factors for poor sleep hygiene practices. Further research is needed to establish the effect of poor sleep quality and inadequate sleep hygiene practices during Ramadan fasting in our region.

## Introduction

The holy month of Ramadan significantly influences the lifestyle of Muslims as it is a unique 30 days in the whole year, and is one of the five pillars of Islam [1]. Since the fasting hours in Saudi Arabia are more than 14 hours, many people are awake all night, reflecting on their routines. However, the change in daily routine significantly affects the sleeping patterns of people. Moreover, Ramadan follows the lunar calendar, occurring in a different season every nine years. As a result, the number of hours spent fasting might vary depending on the season, with summer fasts typically lasting longer than winter fasts [2]. The effect of Ramadan on lifestyle extends to include many aspects, such as working hours, which are shorter and delayed for two or three hours to give people a chance to sleep after the Al-fajr prayer. Additionally, stores and shopping malls accommodate the change by opening in the afternoon and not closing until dawn [3].

Many studies show that bad Ramadan habits are strongly related to poor sleep hygiene, with an impact on sleep patterns. In Saudi Arabia, lifestyles and traditions of this month affect sleep in many aspects. Dietary and lifestyle practices associated with Ramadan vary by country and culture and can affect sleep duration, onset, wakefulness, and sleep disturbances to varying degrees. Thus, sleep and lifestyle changes observed in one country or culture cannot be extrapolated to another [4,5], especially with the increase in the global Muslim population to more than two billion Muslims living in more than 200 countries and territories, accounting for 25% of the world’s population [6].

Studies among the Saudi population focus on the relationship between dietary intake and sleep without focusing on sleep patterns. Hence, in this study, we aim to assess the quality of sleep in the Saudi population during the holy month of Ramadan.

## Materials and methods

This was a cross-sectional study on the quality of sleep among the Saudi adult population during the holy month of Ramadan. Ethical approval was obtained from Imam Mohammad Ibn Saud Islamic University Institutional Review Board (project number: 525/2023). This study focused on both sleep quality and sleep hygiene practices. This was done through an online Google Forms questionnaire distributed through social media. The study sample was all Saudi citizens over the age of 18 who fasted during the holy month of Ramadan and could fill out our questionnaire. Data were collected from all regions of Saudi Arabia (Eastern, Western, Southern, Northern, and Central regions).

The inclusion criteria were adults aged 18 years and above, who fasted during the holy month of Ramadan and were residing in Saudi Arabia. Our exclusion criteria were people below 18 years old, who did not fast during the holy month of Ramadan and were residing outside Saudi Arabia. Our sample size was 386. Our questionnaire included three sections comprising demographic data, a 28-question validated Sleep Quality Scale (SQS), and a validated 13-question Sleep Hygiene Index (SHI). We conducted a pilot study to ensure the clarity of our questionnaire.

The SQS consists of 28 questions, evaluating six domains, namely, daytime symptoms, restorative sleep, problems initiating and maintaining sleep, difficulty waking up, and sleep satisfaction. This scale was formulated by Yi et al. [7]. The scale uses a four-point scoring system indicating the frequency of certain sleep behaviors with 0 = “few,” 1 = “sometimes,” 2 = “often,” and 3 = “almost always.” The scores vary from 0 to 84, with higher scores indicating more acute sleep issues.

The SHI consists of 13 questions; the frequency of certain behaviors is represented by 0 = “never,” 1 = “rarely,” 2 = “sometimes,” 3 = “frequently,” and 4 = “always.” The SHI was developed by Mastin et al. [8]. The scores range from 0 to 52, with higher values denoting an increase in activities that undermine sleep hygiene. A cutoff point of 16 has been used to determine poor sleep hygiene problems [9].

The data were extracted initially from the Google Forms questionnaire into a comprehensively arranged Excel sheet (Microsoft Office 2019 version). The data were analyzed by SPSS version 23 software (IBM Corp., Armonk, NY, USA) and reviewed by a biostatistician.

Statistical analysis

Categorical variables were described as numbers and percentages. Continuous variables were calculated and summarized as mean and standard deviation. The association between the level of SHI and the score of SQS was assessed using the Mann-Whitney Z-test. Additionally, the association between the sociodemographic characteristics in terms of the SQS and SHI scores was determined using the Mann-Whitney Z-test and the Kruskal-Wallis H-test. Both the Kolmogorov-Sminov and the Shapiro-Wilk tests were used to perform normality tests. Both the SQS and SHI scores had a non-normal distribution. Consequently, non-parametric tests were utilized. Statistical significance was set at p < 0.05 level. All data analyses were performed using Statistical Packages for SPSS version 23 software (IBM Corp., Armonk, NY, USA).

## Results

A total of 386 respondents completed the survey. As seen in Table 1, females (71.2%) dominated the study population compared to males (28.8%). Nearly two-thirds (64.5%) were single, and 29% resided in the Central region. Nearly half (47.7%) were students. Overall, 17.9% of the respondents were the main providers for their families. Further, 23.6% were mothers, and 38.3% were living with six to eight family members. Approximately 44.6% slept alone. In addition, 53.6% were earning less than 5,000 SAR per month.

**Table 1:** Sociodemographic characteristics of participants (n = 386).

Study variables	N (%)
Gender	
Male	111 (28.8%)
Female	275 (71.2%)
Marital status	
Single	249 (64.5%)
Married	124 (32.1%)
Divorced or widowed	13 (03.4%)
Residence region	
Central region	112 (29.0%)
Eastern region	46 (11.9%)
Northern region	105 (27.2%)
Southern region	36 (9.3%)
Western region	87 (22.5%)
Occupation	
Student	184 (47.7%)
Employed	149 (38.6%)
Unemployed	22 (5.7%)
Retired	31 (8.0%)
Are you the main provider of the household?	
Yes	69 (17.9%)
No	317 (82.1%)
Are you a parent	
No	261 (67.6%)
Father	34 (08.8%)
Mother	91 (23.6%)
How many individuals in your family/home?	
Alone	7 (1.8%)
1–3	57 (14.8%)
4–6	148 (38.3%)
6–8	129 (33.4%)
9 or more	45 (11.7%)
Do you sleep alone?	
Yes	172 (44.6%)
No, with siblings	106 (27.5%)
No, with spouse	108 (28.0%)
Monthly income (SAR)	
<5,000	207 (53.6%)
5,000–10,000	78 (20.2%)
11,000–15,000	35 (9.1%)
16,000–20,000	37 (9.6%)
>20,000	29 (7.5%)

Table 2 shows the descriptive statistics of sleep quality and sleep hygiene based on the SQS and SHI. It can be observed that the total mean SQS score was 35.8 (SD = 10.9), while the total mean SHI score was 19.8 (SD = 8.61). Accordingly, 63.5% of the respondents were considered to have poor sleep hygiene, while the rest were considered good (36.5%).

**Table 2:** Descriptive statistics of the SQS and SHI (n = 386). SQS = Sleep Quality Scale; SHI = Sleep Hygiene Index

Variables	Mean ± SD
Total SQS score	35.8 ± 10.9
Total SHI score	19.8 ± 8.61
Level of sleep hygiene	N (%)
Poor (score >16)	245 (63.5%)
Good (score ≤16)	141 (36.5%)

**Figure 1:**
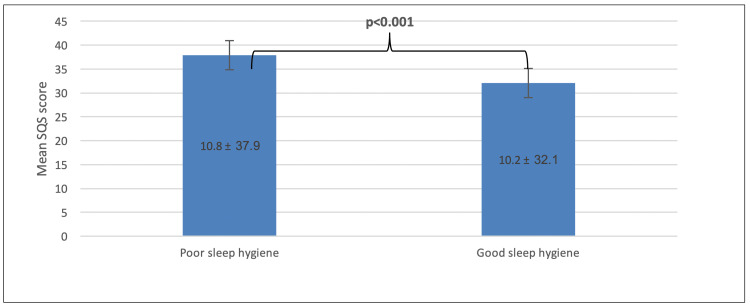
Association between SQS and SHI. SQS = Sleep Quality Scale; SHI = Sleep Hygiene Index

As shown in Figure 1, poor sleep hygiene was more associated with having a higher mean SQS score (p < 0.001).

When measuring the association between the participants’ sociodemographic characteristics regarding SQS and SHI scores, it was found that a higher SQS score was more associated with having more than six individuals at home (H = 7.699; p = 0.021). Regarding SHI, a higher SHI score was more associated with being unmarried (Z = 3.855; p < 0.001), a student (H = 15.297; p < 0.001), not the main provider for the family (Z = 2.120; p = 0.034), not a parent (Z = 4.444; p < 0.001), and earning less than 5,000 SAR monthly (Z = 3.675; p < 0.001) (Table 3).

**Table 3:** Association between the sociodemographic characteristics in relation to the scores of SQS and SHI (n = 386). ^§^: P-value was calculated using the Mann-Whitney Z-test. ^‡^: P-value was calculated using the Kruskal-Wallis H-test. ^**^: Significant at p < 0.05 level. SQS = Sleep Quality Scale; SHI = Sleep Hygiene Index

Factor	SQS score (84); Mean ± SD	Z-test; P-value^§^	SHI score (52); Mean ± SD	Z-test; P-value^§^
Gender				
Male	36.2 ± 10.1	0.636; 0.525	18.5 ± 7.19	1.736; 0.083
Female	35.6 ± 11.3		20.3 ± 9.08	
Marital status				
Unmarried	35.6 ± 10.9	0.188; 0.851	20.9 ± 8.53	3.855; <0.001^**^
Been married	36.2 ± 10.9		17.6 ± 8.34	
Occupation				
Student	35.8 ± 10.9	0.020; 0.990^‡^	21.5 ± 8.67	15.297; <0.001^**^^‡^
Employed	35.8 ± 11.3		18.6 ± 8.59	
Unemployed	35.7 ± 10.1		17.2 ± 7.34	
Are you the main provider of the household?				
Yes	37.4 ± 11.8	1.109; 0.267	17.8 ± 6.95	2.120; 0.034^**^
No	35.4 ± 10.7		20.2 ± 8.88	
Are you a parent				
Yes	36.4 ± 11.3	0.413; 0.680	16.9 ± 7.52	4.444; <0.001^**^
No	35.5 ± 10.8		21.1 ± 8.78	
How many individuals in your family/home?				
3 or less	33.9 ± 12.0	7.699; 0.021^**^^‡^	17.9 ± 8.01	4.465; 0.107^‡^
4 to 6	34.4 ± 10.1		19.3 ± 8.10	
More than 6	37.7 ± 10.9		20.9 ± 9.11	
Do you sleep alone?				
Yes	35.8 ± 11.2	0.113; 0.910	20.4 ± 8.28	1.351; 0.177
No	35.8 ± 10.7		19.3 ± 8.41	
Monthly income (SAR)				
<5,000	36.0 ± 10.7	0.515; 0.607	21.3 ± 8.92	3.675; <0.001^**^
≥5,000	35.5 ± 11.3		18.0 ± 7.90	

## Discussion

According to our study results, 63.5% of the respondents were considered to have poor sleep hygiene, while the rest were considered good (36.5%). Poor sleep hygiene was associated with a higher mean SQS score (p < 0.001). These results are consistent with many previous studies. BaHammam revealed a shift in participants’ sleep habits during Ramadan, with a tendency to go to bed later and wake up earlier. Surprisingly, there was no significant increase in daytime sleepiness despite these changes. Moreover, during the non-fasting period, participants tended to consume fewer meals but more calories [10]. In contrast, Faris et al. indicated an overall small but significant impact of diurnal fasting on sleep patterns. Specifically, there was a slight reduction in total sleep time and an increase in wakefulness after the onset of sleep [11]. This was contrary to a study that indicated that, while there was no significant change in total sleep time or sleep efficiency during Ramadan, there was a substantial increase in wakefulness after sleep onset. The Epworth Sleepiness Scale (ESS) also revealed a considerable rise in daytime sleepiness during the Ramadan fast [12]. Another study showed that there was a large increase in non-rapid eye movement (REM) sleep and a significant decrease in REM sleep during the Ramadan fast. Additionally, the ESS revealed that Ramadan fasting significantly increased daytime sleepiness [13]. A study reported that the duration of sleep was substantially longer before and after Ramadan than it was throughout the holy month. Moreover, there was an increase in the daytime dysfunction score throughout Ramadan. Additionally, the sleep quality score exhibited an increase during both the Ramadan period and the period after, when compared to the period before Ramadan. The sleep disruption score increased during Ramadan compared to before [14]. However, a study that accounted for environmental factors and sleep-wake cycles found no substantial alterations in sleep structure. This suggests that the previously observed changes may be a result of accompanying lifestyle modifications rather than the act of fasting itself. A reduction in the amount of REM sleep is the only constant change across all studies that evaluated sleep architecture during diurnal intermittent fasting (IF) [15]. Another study agreed with these results reporting that night sleep hours were significantly longer both before and after than during Ramadan. Daytime sleep hours pre-Ramadan and post-Ramadan were markedly shorter than during Ramadan [16].

Based on our results, using the SQS questionnaire, the overall mean score was 35.8 (SD = 10.9) out of 84 points. Accordingly, we found that more than half of the respondents (51.0%) scored above the mean, indicating poor sleep quality among our respondents. Several studies have documented a reduction in sleep duration during Ramadan with an increase in daytime sleepiness [1,3,10,11,14,16]. However, a study by Qasrawi et al. [2] revealed that sleep duration, sleep/wake schedule, energy expenditure, and light exposure do not reinforce the notion that IF during Ramadan elevates daytime sleepiness and modifies cognitive function. Similarly, Arora et al. [17] reported that the overall sleep pattern or fatigue levels do not significantly change during Ramadan. Moreover, they reported that eating behaviors and lifestyle modification during Ramadan did not influence daytime sleepiness and functioning.

Having more than six individuals at home was identified as a significant factor for increased SQS score, indicating that the increasing number of family members living in a household was associated with poor sleep quality. However, we found no significant differences between SQS scores in terms of gender, occupation, being the household’s main provider, being a parent, sleeping alone, and monthly income (p > 0.05). In Riyadh [4], one study revealed that smoking significantly affected sleep disturbance and poor sleep quality, while eating vegetables, dates, fruits, and plant-based proteins influenced sleep quality positively. This was supported by a study from Indonesia [18]. Sleep quality showed a significant relationship with food consumption during Ramadan (p < 0.05); however, the relationship between sleep quality and physical activity was insignificant (p = 0.402).

Moreover, we noted a significant correlation between sleep quality and sleep hygiene practices (p < 0.001), suggesting that students with poor sleep hygiene practices tended to have inadequate sleep quality. In Tunisia [14], an increase in scores of daytime dysfunction, sleep quality, and sleep disturbance was observed during Ramadan while sleep efficiency decreased; however, sleep latency had no relevant effect. A study from Malaysia [19] indicated that overall sleep quality significantly improved during Ramadan, followed by sleep latency, sleep efficiency, and sleep disturbances; however, other sleep quality components did not reach statistical significance (p > 0.05).

It is important to highlight that the prevalence of participants with poor sleep hygiene practices was 63.5%. Further, we noted that poor sleep hygiene was more prevalent among unmarried respondents, students, those who were not the household’s main provider, those who were not parents, and those with poor economic status. To our knowledge, this is the first study in Saudi Arabia that compared sleep hygiene practices and the sociodemographic data of the respondents. Hence, more investigations are required to confirm the cause and effect of their associations.

Khan et al. [4] documented an association between sleep duration in terms of physical activity and eating plant-based protein. However, among Polish runners [20], sleep quality and physical performance weakened during Ramadan, and runners who worked besides training displayed the worst-case scenario in terms of physical fitness examination and sleep quality during the month of Ramadan. In contrast, Cahyati et al. [18] found that the association between physical activity and sleep quality during Ramadan was not statistically significant (p > 0.05). In our study, however, we found no significant correlation between sleep hygiene practices concerning demographic data such as gender, number of individuals at home, and sleeping alone (p > 0.05).

The implications of associations of sleep quality with demographic variables are crucial in understanding the factors that contribute to the quality of sleep among different populations. One possible explanation for the existence of associations between sleep quality and demographic variables is the impact of socioeconomic status on access to resources that promote good sleep. Individuals with lower socioeconomic status may have limited access to healthcare, healthy food, and safe living environments, all of which can impact their ability to get quality sleep. Additionally, individuals with lower education levels may be more likely to work in jobs with irregular or demanding schedules, leading to disruptions in their sleep patterns.

Furthermore, cultural differences and societal expectations surrounding sleep may also play a role in shaping the associations between sleep quality and demographic variables. For example, certain cultural norms and expectations may influence individuals’ attitudes toward sleep, leading to differences in sleep behaviors and patterns among different demographic groups.

Another potential explanation for the associations between sleep quality and demographic variables is the impact of stress and discrimination on sleep. Research has shown that individuals from marginalized or minority groups may experience higher levels of stress and discrimination, which can have a detrimental effect on their sleep quality. These experiences may contribute to disparities in sleep quality among different demographic groups.

By understanding the underlying reasons for these associations, researchers and healthcare professionals can develop targeted interventions to improve sleep quality among different demographic groups. This area of research has the potential to have significant public health implications, as improving sleep quality has been linked to numerous health benefits. Future research must continue to explore the complex relationships between sleep quality and demographic variables to develop effective strategies for promoting better sleep for all individuals.

This study is limited by our relatively small sample size. Moreover, as our participants were chosen using convenience sampling and not through randomization, our findings cannot be generalized. The data were collected after the month of Ramadan which may have caused recall bias. 

## Conclusions

Poor sleep quality and unsatisfactory sleep hygiene practices were noted among the Saudi population during the month of Ramadan. Poor sleep quality was more prevalent among those who were living with more than six family members, while lack of sleep hygiene practices was more prevalent among unmarried respondents who were not the main provider of the family and had poor economic status.

This study provides evidence of the adverse effects of Ramadan fasting on sleep quality. Extended daytime sleeping hours could be beneficial to individuals observing Ramadan fasting. It is important to consider the potential long-term health implications of this and explore solutions for mitigating its effects. Adequate sleep is crucial for overall health and well-being, and finding ways to improve sleep quality during this time is essential. It would be beneficial to research and share strategies for optimizing sleep during Ramadan to promote better health outcomes.
